# Generation of Cas9 Knock-In *Culex quinquefasciatus* Mosquito Cells

**DOI:** 10.3390/dna5010001

**Published:** 2025-01-01

**Authors:** Elizabeth Walsh, Tran Zen B. Torres, Brian C. Prince, Claudia Rückert

**Affiliations:** Department of Biochemistry and Molecular Biology, College of Agriculture, Biotechnology & Natural Resources, University of Nevada, Reno, NV 89557, USA

**Keywords:** Cas9, gene editing, gene knock-in, arboviruses, mosquitoes, cell lines, antiviral genes

## Abstract

**Background/Objectives:**

*Culex* species mosquitoes are globally distributed and transmit several pathogens that impact animal and public health, including West Nile virus, Usutu virus, and *Plasmodium relictum*. Despite their relevance, *Culex* species are less widely studied than *Aedes* and *Anopheles* mosquitoes. To expand the genetic tools used to study *Culex* mosquitoes, we previously developed an optimized plasmid for transient Cas9 and single-guide RNA (sgRNA) expression in *Culex quinquefasciatus* cells to generate gene knockouts. Here, we established a monoclonal cell line that consistently expresses Cas9 and can be used for screens to determine gene function or antiviral activity.

**Methods:**

We used this system to perform the successful gene editing of seven genes and subsequent testing for potential antiviral effects, using a simple single-guide RNA (sgRNA) transfection and subsequent virus infection.

**Results:**

We were able to show antiviral effects for the *Cx. quinquefasciatus* genes *dicer-2*, *argonaute-2b*, *vago*, *piwi5*, *piwi6a*, and *cullin4a*. In comparison to the RNAi-mediated gene silencing of *dicer-2*, *argonaute-2b*, and *piwi5*, our Cas9/sgRNA approach showed an enhanced ability to detect antiviral effects.

**Conclusions:**

We propose that this cell line offers a new tool for studying gene function in *Cx. quinquefasciatus* mosquitoes that avoids the use of RNAi. This short study also serves as a proof-of-concept for future gene knock-ins in these cells. Our cell line expands the molecular resources available for vector competence research and will support the design of future research strategies to reduce the transmission of mosquito-borne diseases.

## 1. Introduction

*Culex* mosquitoes are vectors for a variety of pathogens that impact animal and public health, including West Nile virus (WNV) [1], Saint Louis encephalitis virus [2], and Usutu virus (USUV) [3]. *Culex* mosquitoes are also vectors for the parasite *Plasmodium relictum*, which causes avian malaria and has had significant impacts on Hawaii’s native bird populations [4]. Currently, no vaccines are available for these pathogens [5]. Despite their threat to public health, they remain understudied compared to *Aedes* and *Anopheles* mosquito species. Current vector control methods rely on the use of insecticides, but mosquitoes are becoming increasingly resistant to insecticides [6]. New methods that utilize clustered regularly interspaced short palindromic repeat (CRISPR)-associated protein 9 (Cas9) systems have been proposed and tested in several mosquito species to prevent disease transmission or reduce vector population [7]. However, genetic-engineering-based strategies that affect disease transmission require an understanding of proviral and antiviral genes, and the molecular tools to assess gene function in *Culex* species mosquitoes are limited [7].

One avenue to reduce mosquito-borne pathogen transmission would be to manipulate mosquito immune genes via CRISPR/Cas9 editing. In mosquitoes, the antiviral defense is largely mediated by the RNA interference (RNAi) pathways [8]. Dicer-2 (Dcr-2) is responsible for recognizing and cleaving viral double-stranded RNA (dsRNA) into small interfering RNAs (siRNAs), which are then loaded onto the RNA-induced silencing complex (RISC) [9,10]. Argonaute-2 (Ago-2) is a component of RISC and guides the degradation of viral RNA based on the siRNA sequences [11,12]. The loss of Dcr-2 in mosquitoes increases the replication of several arboviruses and insect-specific viruses [13–17]. Similarly, disrupting Ago-2 expression increases virus replication [17–20] and may compromise the balance between mosquito immunity and virus replication. In *Culex quinquefasciatus*-derived cells, another antiviral protein, Vago, is secreted following WNV infection and elicits an antiviral response [21]. However, most of our understanding of antiviral responses in mosquitoes comes from studies performed in *Drosophila melanogaster* flies or *Aedes aegypti* mosquitoes. Less is known in *Culex* species mosquitoes, and fewer molecular tools are available. RNAi has been used for decades to reduce gene expression in mosquitoes and cells, but it is not always very efficient and using an antiviral pathway to study antiviral responses can be confounding. One of our recent studies investigated the effects of silencing multiple *piwi* genes in *Culex quinquefasciatus* cells, where we observed a trend of *piwi5* silencing increasing Usutu virus (USUV) replication, but the effects were minimal and highly variable [22]. Having more efficient methods to reduce gene expression may help to elucidate whether genes such as *piwi5* are, in fact, antiviral.

Recently, studies have validated the feasibility of CRISPR/Cas9 editing in the *Cx. quinquefasciatus* genome, both in mosquitoes through embryo injection or REMOT [23–26], and in cell cultures through plasmid-based methods [27,28]. Briefly, CRISPR/Cas9 systems work via a single guide RNA (sgRNA) directing a Cas9 endonuclease to a complementary DNA target sequence, which Cas9 cleaves to generate a double-stranded DNA break. DNA repair mechanisms are error-prone and can result in insertions, deletions, or mutations that prevent gene expression [29]. We previously optimized a plasmid construct for the transient expression of Cas9 and an sgRNA in *Cx. quinquefasciatus* cells, which enabled the generation of gene knockout cells without constitutive Cas9 expression [27]. In contrast, Feng et al. (2021) used site-directed homology to generate a *Cx. quinquefasciatus* mosquito line expressing Cas9 in germline cells [26]. This mosquito line was made via the injection of a single plasmid that contained Cas9 under the control of the germline-specific *vasa* promoter, a dsRed fluorescent marker, and both flanked by homology regions to the *cardinal* gene locus. Additionally, the plasmid contained a necessary sgRNA cassette for targeted insertion. The mosquito line was found to successfully edit the *kh* gene when an sgRNA was introduced [26].

While Cas9-expressing mosquito lines can be used to understand the genetic mechanisms important to vector competence and disease transmission and will be essential for developing transgenic mosquitoes, administering gRNAs to knockout-specific genes in mosquitos can be technically difficult. Additionally, the upkeep of multiple transgenic mosquito lines requires space and labor. Determining gene function in cell cultures before in vivo validation in mosquito lines can serve as a highly beneficial proof of concept. CRISPR/Cas9 plasmid-based methods have offered an avenue to study gene function in mosquito cell lines. CRISPR/Cas9-edited mosquito cells have been developed previously, including an *Aedes aegypti*-derived Aag2 Dcr-2 knockout cell line, an Aag2 Argonaut 2 (Ago-2) knockout cell line, and an *Aedes albopictus*-derived C6/36 Nix knockout cell line [30–32]. These cell lines were generated through transient expression of Cas9 via plasmid transfection, along with introducing one or more sgRNAs targeting the gene of interest, similar to the plasmid we developed for use in *Cx. quinquefasciatus* cells [27]. In our literature review, we found only one mosquito cell line that stably expresses Cas9, the *Anopheles coluzzi*-derived cell line Sua-5B-IE8-Act::Cas9-2A-Neo, made through recombination-mediated cassette exchange [28]. The stable expression of Cas9 in mosquito cell lines can allow for large-scale genetic screening [28]. Additionally, a stable Cas9-expressing cell line can save on reagents, as only an sgRNA would need to be introduced for genome editing.

In the present study, we developed a *Cx. quinquefasciatus*-derived cell line constitutively expressing Cas9 through the introduction of a single plasmid that contained Cas9 flanked by homology arms to the *cardinal* gene, based on work performed by Feng et al., 2021 [26]. We used a combination of cell sorting and dilution into semi-solid media to generate a monoclonal line, which showed stable expression of Cas9 and successful gene editing of seven genes when single sgRNAs were introduced. We also performed a ‘miniscreen’ using sgRNAs for seven mosquito genes that we hypothesize to be either antiviral or proviral and infection with either the orthobunyavirus La Crosse virus (LACV) or the flavivirus Usutu virus (USUV). We were able to show that six of the chosen genes were antiviral in a virus-dependent manner. This new cell line will be beneficial for studying antiviral mechanisms in *Cx. quinquefasciatus*.

## 2. Materials and Methods

### 2.1. Cell Culture Maintenance

The *Cx. quinquefasciatus*-derived ovarian cell line (Hsu) [33] was kindly provided by Dr. Aaron Brault (CDC for Vector-borne Diseases, Fort Collins). An Hsu-derived monoclonal cell line, named Hsu-Clone21, was generated using flow cytometry as previously described for Aag2 cells [34]. This monoclonal cell line was used to generate the Cas9 knock-in cells. Hsu cells and any Hsu-derived cells were grown at 27 °C and 5% CO_2_ in Dulbecco’s modified Eagle medium (DMEM; Corning #10–013-CV; Corning, NY, USA) supplemented with 10% FBS and 100 units/mL penicillin, 100 μg/mL streptomycin, and 5 μg/mL gentamicin.

### 2.2. Plasmid Transfection

To prepare cells for sorting, Hsu-Clone21 cells were seeded in 6-well plates. Hsu-Clone21 cells at 50–60% confluency were transfected with the plasmid containing vasa-Cas9 flanked by *cardinal* homology regions [26] kindly provided by Valentino Gantz and Víctor López Del Amo. GFP expression under the control of the *vasa* promoter was validated in Hsu cells before the introduction of the Cas9-containing plasmid. Hsu-Clone21 cells were transfected using X-tremeGENE^™^ HP DNA transfection reagent (Roche, #6366236001; Pleasanton, CA, USA) as per the manufacturer’s protocol for a 6-well plate. A 2:1 DNA/transfection reagent ratio was used, and each well received 2 μg of the plasmid.

### 2.3. Keyence Imaging

To validate the successful transfection of the plasmid into Hsu-Clone21 cells, cells were imaged 48 h post-transfection. Aside from vasa-Cas9, the plasmid also contains dsRed under the Opie2 promoter. Prior to imaging, fresh medium was added to the cells, and images of live cells were visualized using a Keyence BZ-X710. Prior to cell sorting, Hsu-Clone21 cells transfected with the Cas9-containing plasmid were moved to 75 cm cell culture flasks (Corning, #734–0965; Corning, NY, USA). Cells were passaged for 5 weeks and imaged weekly to allow for vasa-Cas9 integration into the genome (as opposed to transient expression) prior to cell sorting.

### 2.4. Cell Sorting

Five weeks post-transfection, Hsu-Clone21 cells were seeded into 6-well plates. The medium was removed from Hsu cells at 70–80% confluency, and cells were washed with 2 mL of PBS. After PBS was discarded, 0.5 mL of Accumax (Thermo Fisher, #00-4666-56; Waltham, MA, USA) was added to Hsu cells, and cells were placed in the incubator for 1 min to detach cells from the plate. Cells were centrifuged in a 50 mL conical tube at 500× *g* for 5 min, the medium was removed, and cells were pelleted with 1 mL of sterile sorting buffer (PBS, 25 mM HEPES pH 7.0, 1% FBS heat-inactivated FBS Premium, 2 mM EDTA, 1% Pen-Strep antibiotic), with 1:10,000 DAPI to gate for living cells. Then, 1 mL of cells were filtered into 5 mL tubes through a 25 μm Strainer Cap (Olympics, #28–154; Genessee Scientific, Morrisville, NC, USA). Cells were then sorted for DAPI and dsRed expression using a BD FACSAria II Cell Sorter (Nevada Cytometry Center, Reno, NV, USA). Cells were collected in a 5 mL tube containing 1 mL of DMEM + 20% FBS and kept on ice until approximately 200,000 cells were added to each of 4 wells in a 6-well plate containing 2 mL of fresh pre-warmed DMEM, +20% FBS, and antibiotics. The medium was replaced with fresh medium 24 h post sorting and replaced daily afterward. Once cells were confluent, they were subsequently moved to a 25 cm and 75 cm flask.

### 2.5. Generation of Monoclonal Line Using a Semi-Solid Overlay

Semi-solid sorting was used to develop monoclonal Hsu cells stably expressing Cas9. We have observed better survival using this technique versus single-cell sorting using flow cytometry. Briefly, 45 mL of STEMCELL Technologies ClonaCell^™^ FLEX was added to 40 mL of 2X DMEM, 10 mL of FBS Premium Plus, and 4.44 mL of 7.5% NaHCO3. Cells previously sorted via flow cytometry (i.e., all expressing dsRed) were detached using ACCUMAX (Thermo Fisher, #00-4666-56; Waltham, MA, USA). Cells were then filtered into 5 mL tubes through a 25 μm Strainer Cap (Olympics, #28–154; Genessee Scientific, Morrisville, NC, USA), and cells were counted using a hemocytometer. The cell suspension was diluted and added to 6-well plates containing 2.5 mL of STEMCELL Technologies ClonaCell^™^ FLEX mixture at a concentration of 25 cells/well. Hsu cells were incubated at 27 °C in a humidified, 5% CO_2_ incubator. Once distinct colonies expressing dsRed formed, they were removed using a P-1000 pipette tip and transferred to a 24-well plate containing fresh DMEM, +20% FBS, and antibiotics. Cells were expanded and aliquots of cells were frozen using liquid nitrogen for storage.

### 2.6. PCR

To verify the integration of Cas9 into the *Cx. quinquefasciatus* genome, PCR primers were designed to amplify from 252 bp outside of the right flanking homology arm within the *cardinal* gene to inside the Cas9 endonuclease gene (Table 1). Briefly, genomic DNA was extracted from our monoclonal dsRed-expressing cells as well as mock-transfected Hsu cells (negative control) using the Zymo Quick-DNA ^™^ Miniprep kit (Zymo Research, # D3024; Irvine, CA, USA) as per the manufacturer’s protocol. DNA was quantified using a Qubit Flex Fluorometer (Thermo Fisher, Waltham, MA, USA), normalized to 10 ng/μL. Target regions were amplified by PCR using Platinum^™^ SuperFi II PCR Master Mix (Thermo Fisher, #12368010 Waltham, MA, USA). DNA was quantified using a Qubit Flex Fluorometer (Thermo Fisher, Waltham, MA, USA), normalized to 10 ng/μL, and verified with gel electrophoresis.

Further, to validate Cas9 expression, RNA was extracted from our Cas9 knock-in Hsu cells five weeks after single-cell generation, as well as mock-transfected Hsu cells using the Directzol RNA miniprep kit (Zymo Research, #D4033; Irvine, CA, USA), as per the manufacturer’s protocol. RNA was quantified using a Qubit Flex Fluorometer (Thermo Fisher, Waltham, MA, USA). Following RNA extraction, cDNA was made using the High-Capacity cDNA reverse transcription kit (Thermo Fisher, #4368814, Waltham, MA, USA). PCR primers were designed to amplify a short region within Cas9 (Table 1) to validate mRNA expression. The target region was amplified by PCR using Platinum^™^ SuperFi II PCR Master Mix (Thermo Fisher, #12368010 Waltham, MA, USA), and DNA was normalized to 10 ng/μL using a Qubit Flex Fluorometer (Thermo Fisher, Waltham, MA, USA), and verified through gel electrophoresis.

### 2.7. sgRNA Design and gRNA Transfection

CRISPR GuideXpress (https://www.flyrnai.org/tools/fly2mosquito/web, accessed on 1 March 2021) was used to design the single guide RNAs (sgRNAs) targeting the *Cx. quinquefasciatus* genomic loci of *dcr-2*, *ago2b*, *vago*, *piwi5*, *piwi6a*, *spcs1*, and *cullin4a* (Table 2). The sgRNAs with the highest Housden efficiency score, an off-target score of 0, and coverage of <60% were chosen and obtained by custom order from Synthego (Redwood City, CA, USA). The efficiency of these gRNAs was validated in previous experiments using transient Cas9 transfection. Monoclonal Cas9 knock-in Hsu cells were seeded into 24-well plates prior to transfection. Single gRNAs were transfected into Cas9 knock-in Hsu cells using Lipofectamine^™^ RNAiMAX (Thermo Fisher Scientific, #13778150; Waltham, MA, USA) according to the manufacturer’s protocol. Briefly, 21.5 μL of Opti-MEM with 2 μL of Lipofectamine^™^ RNAiMAX was combined with 21.5 μL of Opti-MEM with 17.5 μM sgRNA. The lipoplex was incubated for 10 min at room temperature before addition into wells containing 0.5 mL fresh DMEM, +10% FBS, and antibiotics. Transfected cells were incubated at 27 °C and 5% CO_2_.

### 2.8. T7 Assay

A T7 endonuclease assay was used to assess gene editing efficiency as previously described [27,35]. The extent of cleavage by T7 endonuclease corresponds to the degree of gene editing in the cell population. Genomic DNA (gDNA) was extracted three days post sgRNA transfection using the Quick-DNA Miniprep kit (Zymo Research). The target loci were PCR-amplified with primers flanking the edited region (Table 3), using Q5^®^ Hot Start High-Fidelity Master Mix (New England Biolabs, #E2621; Ipswich, MA, USA) and 100 ng of gDNA in a 50 μL reaction. PCR products were visualized on agarose gel electrophoresis, and the bands (900–1000 bps) representing the sgRNA-target regions were isolated using a Zymoclean Gel DNA Recovery kit (Zymo Research, #D4002; Irvine, CA, USA). For the T7 endonuclease I assay, 200 ng of gel-purified PCR amplicons were mixed with 10× NEBuffer 2 (New England Biolabs, #B7002; Ipswich, MA, USA) and denatured at 95 °C for 5 min, followed by gradual cooling to form potential heteroduplexes. Then 1 μL of T7 Endonuclease I enzyme (New England Biolabs, #M030; Ipswich, MA, USA) was added, and the mixture was incubated at 37 °C for 15 min. The reaction was stopped with 1.5 μL EDTA, and the resulting products were separated by gel electrophoresis on a 1% agarose gel in parallel with the 1 kb Plus DNA ladder (New England Biolabs, #N3200; Ipswich, MA, USA).

### 2.9. Gene-Specific dsRNA Synthesis

Gene-specific dsRNA was synthesized using the MEGAScript^™^ RNAi Kit (Thermo Fisher, #AM1626, Waltham, MA, USA) according to the manufacturer’s protocol. Briefly, PCR primers containing T7 promoter sequences (Table 4) were designed to amplify the *ago2b* gene from Hsu cDNA, which was synthesized using the High-Capacity cDNA Reverse Transcription Kit (Thermo Fisher, #4368814, Waltham, MA, USA). For the non-specific control, GFP dsRNA was synthesized using a GFP-containing plasmid and T7 primers (Table 1). PCR amplification of target regions was performed using Q5^®^ Hot Start High-Fidelity Master Mix (New England Biolabs, #M0494) and verified by gel electrophoresis. PCR products were purified and concentrated using the Zymo DNA Clean & Concentrator^™^−25 Kit (Zymo Research, #D4033, Irvine, CA, USA). The purified DNA was transcribed into dsRNA in vitro using the MEGAScript^™^ RNAi Kit (Thermo Fisher, #AM1626, Waltham, MA, USA).

### 2.10. Transfection of dsRNA and siRNA

Hsu cells were seeded into 24-well plates with 500 μL of culture medium per well. After overnight incubation at 27 °C to facilitate cell adhesion, transfections were performed using gene-specific dsRNA and a non-specific dsRNA control (GFP). For *dcr-2* silencing we used a gene-specific siRNA (GAAGAAGUACCUUCUCUACAAGGAA) compared to a *Renilla luciferase* siRNA control (AGAAGUUCCCUAACACCGAGUUCGU), which were both purchased from Integrated DNA Technologies (Coralville, IA, USA) as dsiRNAs. Lipofectamine^™^ RNAiMAX Transfection Reagent (Thermo Fisher, #13778075, Waltham, MA, USA) was used as per the manufacturer’s protocol. Specifically, 1.5 μL of Lipofectamine^™^ RNAiMAX reagent (Thermo Fisher, #13778100, Waltham, MA, USA) was diluted in 25 μL of Opti-MEM^™^ Reduced-Serum Medium (Thermo Fisher, #31985062, Waltham, MA, USA) per well. Separately, 500 ng of dsRNA or 5 pmol siRNA was diluted in 25 μL of Opti-MEM^™^ per well. The diluted dsRNA or siRNA was then mixed with the diluted Lipofectamine reagent and incubated at room temperature for 15 min. Then, 50 μL of the resulting dsRNA-Lipofectamine complex was added to each well, followed by 450 μL of fresh complete culture medium. The cells were incubated for 48 h and then infected with either LACV or USUV.

### 2.11. Virus Infection

Hsu cells were seeded in 24-well plates at a density of 2 × 10^5^ cells per well and infected with either LACV at a multiplicity of infection (MOI) of 10 or USUV at an MOI of 50. The virus was diluted in 250 μL of culture medium (DMEM or Schneider’s medium without additives) per well and added to cells after the existing complete medium was removed. Following a 2 h incubation at 27 °C, the virus-containing medium was aspirated, and 500 μL of complete culture medium was added to each well. Plates were incubated at 27 °C for 48 h, and cells were lysed as per the manufacturer’s instructions; RNA extraction was performed using the Directzol RNA miniprep kit (Zymo Research, #D4033; Irvine, CA, USA).

### 2.12. Quantitative Reverse-Transcriptase qRT-PCR

qRT-PCR was conducted using the primers in Table 4 and the iTaq^™^ Universal SYBR^®^ Green One-Step Kit (Bio-Rad, #1725150, Hercules, CA, USA) or the iTaq^™^ Universal Probes One-Step Kit (Bio-Rad, #1725141, Hercules, CA, USA) on a CFX96 Touch^™^ Real-Time PCR Detection System (Bio-Rad, Hercules, CA, USA). Gene or viral RNA levels were normalized to the previously validated housekeeping gene actin-5c, or virus standards were used to determine virus levels.

## 3. Results

### 3.1. Hsu Derived Monoclonal Cell Line That Stably Expresses Cas9

**Figure 1.**
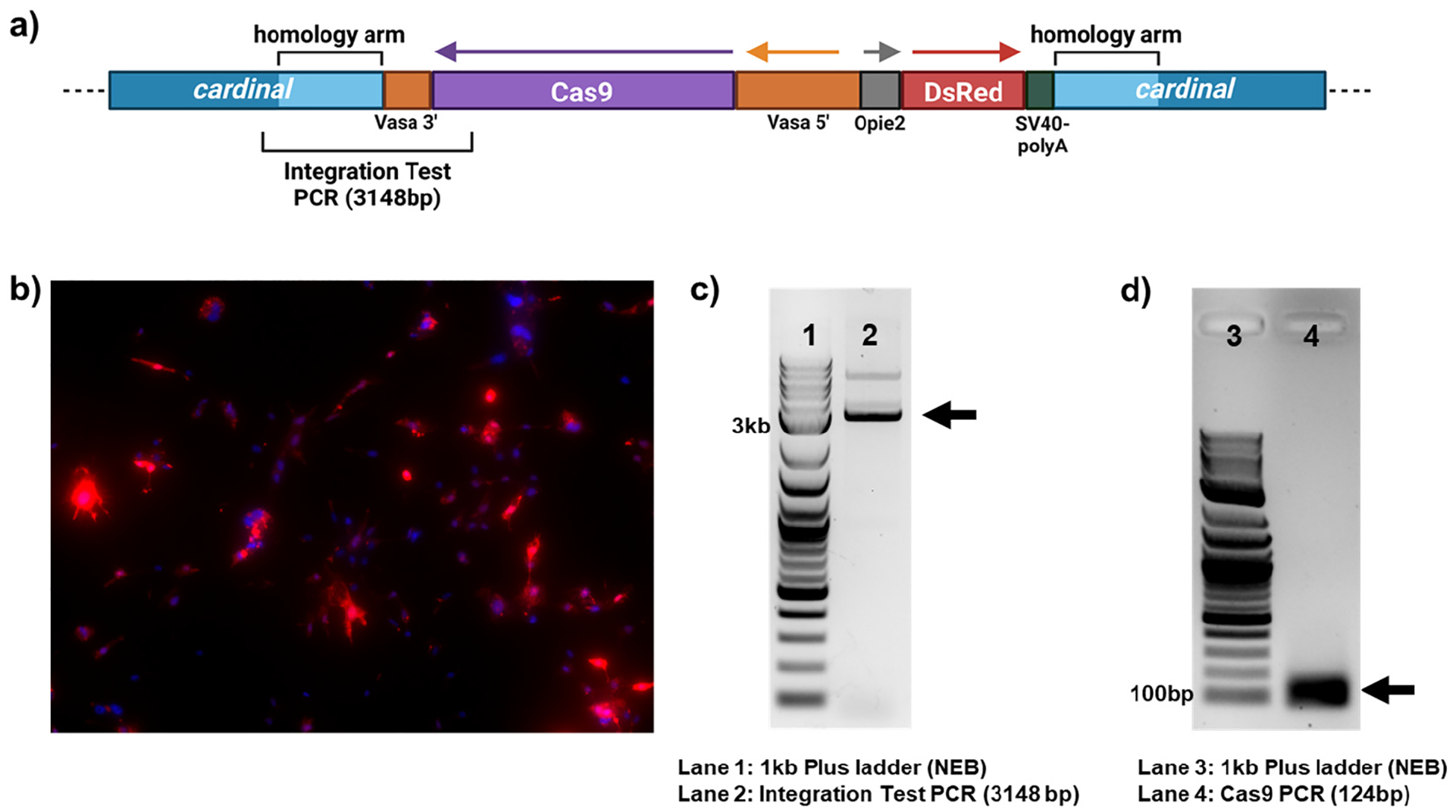
Cas9 integration and expression in Cas9 knock-in Hsu cells. (**a**) Schematic of integrated vasa-Cas9 construct [26]. (**b**) Detection of dsRed by fluorescence microscopy in Cas9 knock-in Hsu cells. Cells were stained with DAPI. (**c**) A 3148 bp PCR product was produced from genomic DNA of Cas9 knock-in Hsu cells, with PCR primers designed to amplify the region flanking the integration site (3148 bp product), visualized here on a 1% agarose gel. (**d**) RT-PCR was performed on RNA of Cas9 knock-in Hsu cells, with PCR primers designed to amplify a region of *cas9* mRNA. Visualized here on a 1% agarose gel. Black arrows in (**c**,**d**) indicate the anticipated PCR product of the correct size.

To develop an Hsu cell line that stably expressed Cas9, we utilized the plasmid developed by Feng et al., 2021 [26]. This plasmid contains the *cas9* gene under the control of the germline-specific *vasa* promoter. Additionally, the plasmid contains *dsRed* under the control of an *opie2* promoter for plasmid integration visualization, flanked by ~1500 bp homologous regions to the *Cx. quinquefasciatus cardinal* gene (Figure 1a). Following plasmid transfection, flow cytometry, and the generation of monoclonal cells using a semi-solid overlay, we observed *dsRed* expression in all cells (i.e., 10 weeks post-transfection), but with varying levels of expression (Figure 1b). This expression 10 weeks post transfection indicates that we successfully generated stable cell lines, possibly with genome integration of the construct. To further verify *cas9* integration into the genome, we used PCR to amplify a region flanking the 5′ integration site. We used primers binding in the *cardinal* gene outside of the homology region and primers binding within *cas9* to amplify DNA across the integration site (‘Integration Test PCR’ in Figure 1a). We found that *cas9* was integrated successfully into the *cardinal* locus of the genome, as indicated by a distinct band of 3148 bp (Figure 1c).

While we were able to detect *dsRed* expression and successful genomic integration, we further needed to verify that Cas9 is expressed, since *dsRed* is controlled by a separate *opie2* promoter. We readily detected *cas9* mRNA using RT-PCR (Figure 1d) and decided to proceed with functional validation.

### 3.2. Cas9 Knock-In Hsu Cells Show Strong Gene-Editing Capabilities

**Figure 2.**
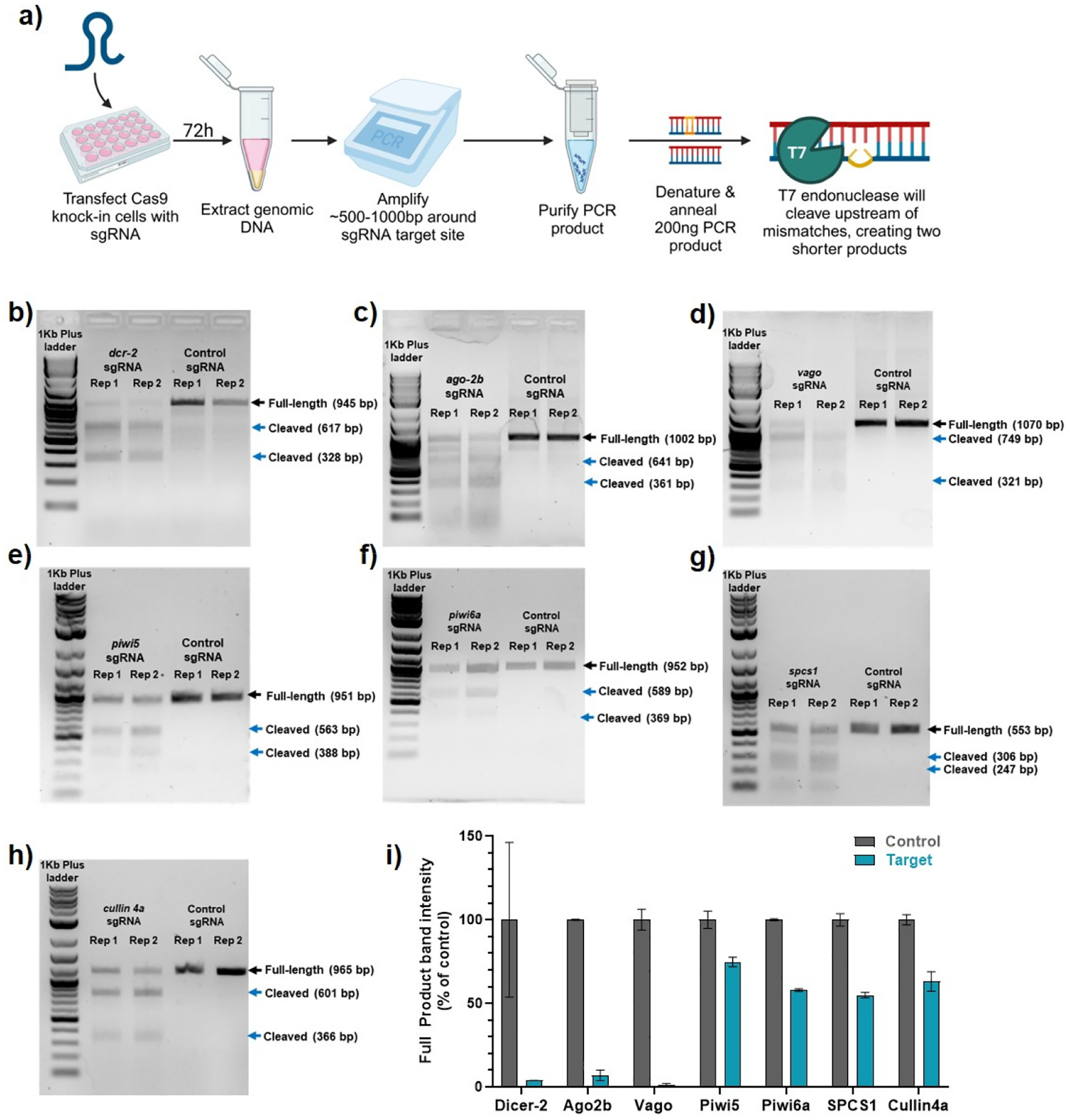
Evaluation of gene editing in Cas9 knock-in Hsu cells. Cas9 knock-in Hsu cells were transfected with a gene-specific sgRNA and a non-targeting control sgRNA. Three days post-transfection, a T7 endonuclease I assay was used to determine CRISPR/Cas9 activity. (**a**) Schematic of experimental design. T7 cleavage after targeting genomic loci of *dcr-2* (**b**), *ago-2b* (**c**), *vago* (**d**), *piwi5* (**e**), *piwi6a* (**f**), *spcs1* (**g**), and *cullin4a* (**h**). PCR products were visualized using a 1% agarose gel and a semi-quantitative assessment was performed by quantifying the signal intensity of the full-length uncut product in ImageJ (**i**).

To verify the gene-editing capabilities of the integrated and expressed Cas9 protein, we administered sgRNAs to edit several known antiviral genes, as well as genes that we suspect to be antiviral or proviral based on the literature [17,21,22,36,37]. We first transfected a synthetic sgRNA that targets exon three of *Cx. quinquefasciatus dcr-2* in parallel with a non-specific sgRNA as a control, as previously used for *dcr-2* editing with transient Cas9 expression [27]. A T7 endonuclease assay was used to assess gene editing efficiency [35]. This assay works by detecting and cleaving heteroduplexed DNA, such as those formed from denatured and re-annealed PCR products of a mixed population of CRISPR/Cas9 edited and non-edited cells (Figure 2a). The extent of cleavage by T7 endonuclease corresponds to the degree of heterogeneity at the locus, providing a semi-quantitative assessment of gene editing in the cell population. We observed no significant amount of T7 cleavage in our control sgRNA transfected cells (Figure 2b). A single band of 945 bp was observed (Figure 2b). However, we detected significant cleavage of the *dcr-2* PCR product following *dcr-2* sgRNA transfection in both replicates (Figure 2b).

We observed products of the expected size for T7 cleavage (617 bp and 328 bp) with minimal residual uncleaved PCR product in samples transfected with *dcr-2* sgRNAs, suggesting high sequence variation at this site, indicative of gene editing (Figure 2b). Similar T7 cleavage results were seen for cells transfected with sgRNAs targeting *ago2b* (Figure 2c), *vago* (Figure 2d), *piwi5* (Figure 2e), *piwi6a* (Figure 2f), *spcs1* (Figure 2g), and *cullin4a* (Figure 2h). We used ImageJ to quantify the band intensity (pixel count) of the full-length PCR product to provide a semi-quantitative assessment of T7 cleavage (Figure 2i). Based on the average of the two replicates, we found that T7 endonuclease cleaved over >95% of *dcr-2* sgRNA-targeted products, >89% of *ago-2* sgRNA-targeted products, and >97% of *vago* sgRNA-targeted products, but less of the other four targeted products, assessed in a separate experiment (Figure 2i). Overall, the cell line exhibited good CRISPR/Cas9 activity with more reliable editing compared to our previous editing experiments using transient plasmid-based expression of Cas9 and sgRNAs [27]. However, some differences between ‘batches’/experiments and genes were observed.

### 3.3. Cas9 Knock-In Hsu Cells Can Be Used to Screen Genes for Antiviral or Proviral Activity

**Figure 3.**
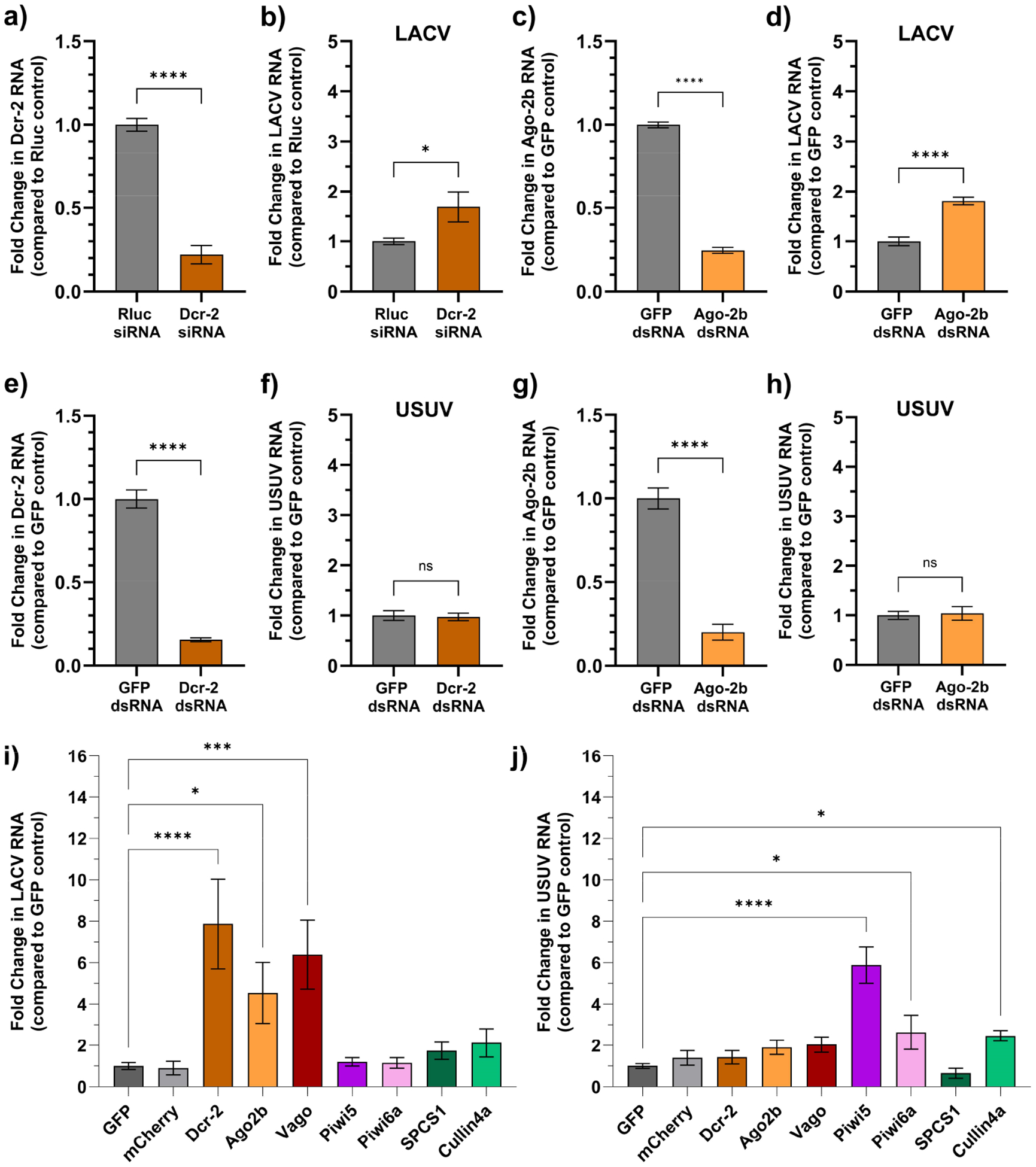
Cas9 knock-in Hsu cells for antiviral screens. First, wildtype Hsu cells were transfected with either siRNA targeting *dcr*-2 (**a**,**b**) or dsRNA targeting *ago-2* (**c**,**d**) and infected with LACV (MOI 10) 48 h post transfection. Gene silencing was validated (**a**,**c**) and LACV RNA quantified (**b**,**d**) by qRT-PCR 48 h post infection. Then, wildtype Hsu cells were transfected with dsRNA targeting *dcr*-2 (**e**,**f**) or *ago-2* (**g**,**h**) and infected with USUV (MOI 50) 48 h post transfection. Gene silencing was validated (**e**,**g**) and USUV RNA quantified (**f**,**h**) by qRT-PCR 48 h post infection. Cas9 knock-in Hsu cells were transfected with sgRNAs targeting seven different genes and two controls (GFP and mCherry). Then, 72 h later, cells were infected with either LACV at MOI 10 (**i**) or USUV at MOI 50 (**j**). RNA was extracted 48 h post infection, and LACV (**i**) or USUV (**j**) RNA was quantified using qRT-PCR and normalized to the GFP control. Mean values from at least two separate experiments with three replicates each are shown. Error bars indicate SEM. Statistical significance was determined using one-way ANOVA and is indicated as ns means non-significant, * *p* < 0.05, *** *p* < 0.0005, and **** *p* < 0.0001.

One of our long-term goals is to identify and study mosquito genes that impact virus replication (antiviral/proviral). The most common method for the silencing of gene expression in mosquitoes is RNAi using either siRNA or dsRNA transfection. However, RNAi is in itself an antiviral pathway in mosquitoes, meaning that RNAi-based gene silencing inherently carries a risk of confounding factors. We conducted ‘traditional’ gene silencing experiments using siRNA or dsRNA to determine whether *dcr-2* or *ago2b* silencing results in increased LACV or USUV replication in Hsu cells. We found that despite a 78% reduction in *dcr-2* mRNA levels following siRNA silencing (Figure 3a), there was only a modest 1.7-fold increase in LACV RNA (*p* < 0.05). Similarly, following a 75% reduction in *ago2b* mRNA using dsRNA silencing, LACV levels were modestly increased (Figure 3b) by 1.8-fold (*p* < 0.0001). There was no increase in USUV replication following the silencing of *dcr-2* (Figure 3e,f) or *ago-2* (Figure 3g,h).

An alternative for RNAi would be to use sgRNAs in our Hsu knock-in cell line to generate partial knock-out cells that will have reduced gene expression. We wanted to test whether a partial knock-out using sgRNA transfection can be used to screen for antiviral or proviral genes. The transfection of sgRNAs into our Cas9 knock-in cells may never knock out genes completely in the whole culture, but if enough cells/alleles are edited, a large proportion of cells may have reduced or no expression of the target gene. We performed a ‘mini-screen’ targeting the seven genes used above (*dcr-2*, *ago2b*, *vago*, *piwi5*, *piwi6b*, *spcs1*, and *cullin4a*) to test for potential antiviral or proviral activity. We transfected Cas9 knock-in Hsu cells with sgRNAs and infected cells 3 days later with either LACV or USUV, representing arboviruses from two different virus families, *Peribunyaviridae* and *Flaviviridae*, respectively.

We observed a significant increase in LACV RNA in cells transfected with *dcr-2* (7.9-fold; *p* < 0.0001), *ago2b* (4.5-fold; *p* < 0.05), and *vago* (6.4-fold; *p* < 0.0005) sgRNA, but no significant differences following sgRNA transfections targeting the other four genes (Figure 3i). In addition, we observed a significant increase in USUV RNA in cells transfected with *piwi5-2* (5.9-fold; *p* < 0.0001), *piwi6a* (2.6-fold; *p* < 0.05), and *cullin4a* (2.5-fold; *p* < 0.05) sgRNA, but again no significant differences following sgRNA transfections targeting the other four genes (Figure 3j). The only gene that had no significant impact on either virus was *spcs1*, but the transfection of *spcs1* sgRNA showed a non-significant trend of reducing USUV replication by 34%.

## 4. Discussion

In this study, we successfully developed a stable Cas9-expressing cell line derived from a monoclonal *Cx. quinquefasciatus* Hsu cell line, providing a valuable tool to study gene function in this understudied mosquito species. Our results demonstrated Cas9 activity and gene-editing of seven genes that are of interest as candidate antiviral or proviral genes. Using these Cas9 knock-in cells, editing of the *dcr-2* locus, specifically, was more apparent compared to our previous plasmid-based transient expression of Cas9 and sgRNA [27]. This gene editing following a simple transfection of sgRNAs into our cell line can facilitate functional studies of mosquito immune genes to understand vector competence and viral interactions. We performed such an experiment and were able to show antiviral activity for six *Cx. quinquefasciatus* genes. Specifically, we showed antiviral activity for *dcr-2*, *ago2b*, and *vago* during LACV infection and *piwi5*, *piwi6a*, and *cullin4a* during USUV infection. To the best of our knowledge, this study is also the first gene knock-in in Hsu cells and acts as a proof-of-principle for future knock-ins of exogenous genes in this cell line, such as dCas9 [38], dCas9-VPR [39], and Cas13 [40], to increase our molecular toolkit or other genes to further study antiviral responses in vitro.

Viswanatha et al., 2021, generated an *Anopheles coluzzii* cell line with stable Cas9 expression using recombination-mediated cassette exchange. This cell line was used to perform large-scale CRISPR screens to study gene function [28]. By integrating Cas9 into the genome, the authors eliminated the need for repeated transfections, which is often a limitation in transient expression systems. A similar approach could be used for the *Cx. quinquefasciatus* Cas9 knock-in Hsu cell line developed in our study to understand genes related to immunity and vector competence before performing in vivo experiments in *Cx. quinquefasciatus* mosquitoes.

Using seven select sgRNAs, we performed a ‘mini-screen’ and made a few interesting discoveries. We found that the key components of the RNAi machinery Dcr-2 and Ago-2 are antiviral against LACV but not USUV in Hsu cells. We observed the same result with RNAi-mediated gene silencing, but the impacts on LACV replication were more pronounced in our Cas9-mediated screen compared to RNAi, suggesting a more robust tool to identify antiviral genes. We also observed that Vago was antiviral against LACV, but not USUV. This was interesting for us to see, because Vago has previously been implicated in antiviral responses to WNV, a virus closely related to USUV. In contrast, the editing of two *piwi* genes, *piwi5* and *piwi6a*, had no effect on LACV replication, but significantly increased USUV replication. We previously observed a trend for antiviral activity of *piwi5* against USUV using RNAi-mediated gene silencing, but only observed a very modest increase and no statistical significance [22]. This highlights again the increased sensitivity of this type of screen compared to RNAi in Hsu cells.

Finally, we included two genes that we thought may have proviral activity, *spcs1* and *cullin4a*, based on previous studies showing that the SPCS1 protein is required for flavivirus assembly in mammalian cells [37] and that the ubiquitin ligase Cullin4 is a proviral factor in *Cx. quinquefasciatus* mosquitoes and cells during WNV infection [36]. We observed a non-significant trend for proviral activity of SPCS1 during USUV infection, but no effect on LACV replication. Since we only measured RNA replication here and did not perform infectious virus titrations, it is possible that any proviral effect of SPCS1 would be more pronounced and statistically significant on virus titers due to its role in virus assembly [37]. SPCS1 in mammalian cells has no antiviral or proviral effects on bunyaviruses, so it is unsurprising that LACV was not affected by *spcs1* editing. The other hypothesized proviral protein, Cullin4, is a cullin RING ubiquitin ligase that degrades the signal transducer and activator of transcription (STAT) and thus blocks immune responses associated with JAK/STAT signaling [36]. It was shown that the silencing of *cullin4a* gene expression reduced WNV replication and overexpression increased WNV replication [36]. However, we observed an increase in USUV replication following transfection of a *cullin4a*-targeting sgRNA. While unexpected, we also showed that USUV is not affected by the secreted cytokine *vago* (unlike WNV), which activates JAK/STAT signaling in *Cx. quinquefasciatus* mosquitoes [21], suggesting that JAK/STAT signaling has no effect on USUV. How *Cx. quinquefasciatus* Cullin4 mediates antiviral activity against USV remains unknown. Follow-up in vivo studies would be required to shed further light on the impacts of Cullin4 on USUV in vivo. Overall, our results demonstrate the use of this Cas9 knock-in cell line and how antiviral immune responses may have unique effects on different virus–vector combinations.

Using a similar workflow to the present study, future work will focus on generating a non-cleaving Cas9 (dCas9) and dCas9-VPR-expressing Hsu cell line, allowing transcriptional control of endogenous genes. Similar dCas9 systems have been utilized in *Aedes albopictus* C6/36 cells and *Aedes aegypti* mosquitoes as a CRISPR activation system to upregulate genes of interest [41,42]. In *Drosophila*, dCas9 has been used for CRISPR interference, for example, to reduce the transcription of long noncoding RNAs [43]. We have shown that the *cardinal* gene serves as a site for integration and successful gene expression in Hsu cells and could be used for the knock-in of a variety of genes, including dCas9 and dCas9-VPR. Overall, developing a dCas9-based system in *Cx. quinquefasciatus*-derived cells will provide a versatile approach to studying gene function and regulation.

The limitations of any gene editing or gene silencing approach include poor annotations of the *Cx. quinquefasciatus* genome, genetic variation between mosquito colonies, and the resulting need to sequence genome loci of interest to ensure that there are no mismatches in sgRNA target sites. This is not unique to sgRNAs, but we have come across sequence variations (SNPs, but also indels) in our cell lines that do not match the NCBI/VectorBase-annotated sequence, resulting in sgRNA designs that cannot cleave as anticipated. However, this is also a concern when using siRNAs, which may not allow multiple mismatches to mediate gene silencing. A recent assembly of the *Culex quinquefasciatus* genome may help the development of robust sgRNAs, but also highlights high variation between *Cx. quinquefasciatus* genomes [44]. Poor genome annotation may be most challenging for the future design of CRISPRa technology due to the need for sgRNAs that bind in noncoding regions (promoter regions), which may be annotated even less reliably than coding sequences. Hopefully, as molecular methodologies and research develop further, genome annotations will improve as well, but natural variation between mosquito colonies will always have to be taken into consideration.

In summary, we developed a Cas9 knock-in Hsu cell line that provides an efficient platform for genome editing. Our cell line expands the current toolkit and will facilitate the future study of *Cx. quinquefasciatus* immune genes and genes related to vector competence. The present study provides a new tool to facilitate the development of genetic strategies to combat mosquito-borne diseases.

## Figures and Tables

**Table 1.** Primers used for C as9 PCRs.

Name	Primer Sequence (5′–3′)
*cas9*-genome-integration-F	GTGAACGAAACTCAGGTGTG
*cas9*-genome-integration-R	CTGGGTATCACGATCATGGA
*Cas9*-cDNA-F	GGATCGACTCCTTCGAGAAAC
*Cas9*-cDNA-R	GGTGAGATCGTGTGGGATAAG

**Table 2.** sgRNAs used for gene editing.

Gene	Oligonucleotides (5′–3′)
*gfP*	GUCGCCCUCGAACUUCACCU
*mCherry*	CAACGAGGACUACACCAUCG
*dicer-2* (CPIJ010534)	CCCGACAGGCCAAUCACCCG
*ago-2b* (CPIJ009898)	UCCGGCAUGAAGAUCGACAA
*vago* (CPIJ012666)	GGCAGCCAAGGAGCCACCAA
*piwi5* (CPIJ017382)	UUCGCACGGGAUACCAACAU
*piwi6a* (CPIJ017381)	CGUCUCCAUAUAGACCACCG
*spcs1* (CPIJ005783)	GGCGCCGUCGGACUGGUCUG
*cullin4a* (CPIJ010574-RA)	ACACCGGAACCGGGAAGCCC

**Table 3.** Primers used for T7 assays.

Name	Primer Sequence (5′–3′)
T7-Assay_*dcr2*_F	CAAGCGCACCTTCTTCATCGTG
T7-Assay_*dcr2*_R	GCTTTGATCGACGAAAACAGCG
T7-Assay_*ago2b*_F	CCGGAGGTGGAGGAGGAGG
T7-Assay_*ago2b*_R	GCCGCTTCCTTCATCGTAATGG
T7-Assay_*vago*_F	TCAGTTATCAGCAATATTTC
T7-Assay_*vago*_R	TATTTTGACTGAATGTATTG
T7-Assay_*piwi4*_F	GAGCACCGAAACGACCGAT
T7-Assay_*piwi4*_R	GTGATCGAAGAAGCGCGTGTT
T7-Assay_*cullin4a*_F	CGTCGTCCTCAGTTCGGAAG
T7-Assay_*cullin4a*_R	CGCTTCAGGATTTTGGCACT
T7-Assay_*spcs1*_F	GCATCTGTCAAACATCCGAGA
T7-Assay_*spcs1*_R	GTTCCGATCCTGGGGTTGTG
T7-Assay_*piwi5*_F	CAGCGACGAGGTCGAGTATC
T7-Assay_*piwi5*_R	GCCCGCTTCTTACTTGGTCT
T7-Assay_*piwi6a*_F	GAGATGAGGCGTGATTTTCAGTA
T7-Assay_*piwi6a*_R	CGGGTGGTAACGGTGCTTAC

**Table 4.** Primers used for virus infection experiments.

Name	Primer Sequence (5′–3′)
Ago-2b dsRNA_F	TAATACGACTCACTATAGGGGCCGTTGGTTTTGGCGTTGAT
Ago-2b dsRNA_R	TAATACGACTCACTATAGGGTTCTGGTTACGGCCTTCGAC
GFP dsRNA_F	TAATACGACTCACTATAGGGATGGTGAGCAAGGGCGAGGAGCTGTTC
GFP dsRNA_R	TAATACGACTCACTATAGGGCTGGGTGCTCAGGTAGTGGTTGTCGGGC
qPCR Dicer-2—R	GATGCAAGGGCTGGAGATAAA
qPCR Dicer-2—F	CTGCGACCTTCCTTGTAGAAC
qPCR Ago2b—F	CCTCGCTGAACCGTAATTCT
qPCR Ago2b—R	AGGTCCAGTGTAAACCACTTC
qPCR Actin 5c—F	CAACTGCCCAAATCGAATGAC
qPCR Actin 5c—R	CGACGCACTCTCGGAATAAA
qPCR LACV—F	CAGCCCAGACAGCCATAAA
qPCR LACV—R	CCCTGGTAGCATGTTGTATGT
qPCR LACV_probe	CCATGCTAGACTGGGTGGACAACC
qPCR USUV—F	CATCAAGGTTCTCTGCCCATAC
qPCR USUV—R	GAAAGAGGGACTCGAACCAATC
qPCR USUV_probe	AGCGCTTGGAAGTTCTACAACGGA

## Data Availability

Cell line can be made available upon request.
